# Supplementary material for: IFI16 Induced by Direct Interaction between Esophageal Squamous Cell Carcinomas and Macrophages Promotes Tumor Progression via Secretion of IL-1α
Source: Cells. 2023 Nov 10;12(22):2603. doi: 10.3390/cells12222603 (PMC10670642; doi:10.3390/cells12222603)
Supplement: Supplementary file 1 [file cells-12-02603-s001.zip › Supplementary Materials_IFI16_for_Cells_final.pdf]

Supplementary Materials

IFI16 Induced by Direct Interaction between Esophageal Squamous Cell Carcinomas and Macrophages Promotes Tumor Progression via Secretion of IL-1α

Yuki Azumi, Yu-ichiro Koma, Shuichi Tsukamoto, Yu Kitamura, Nobuaki Ishihara, Keitaro Yamanaka, Takashi Nakanishi, Shoji Miyako, Satoshi Urakami, Kohei Tanigawa, Takayuki Kodama, Mari Nishio, Manabu Shigeoka, Yoshihiro Kakeji, Hiroshi Yokozaki

A

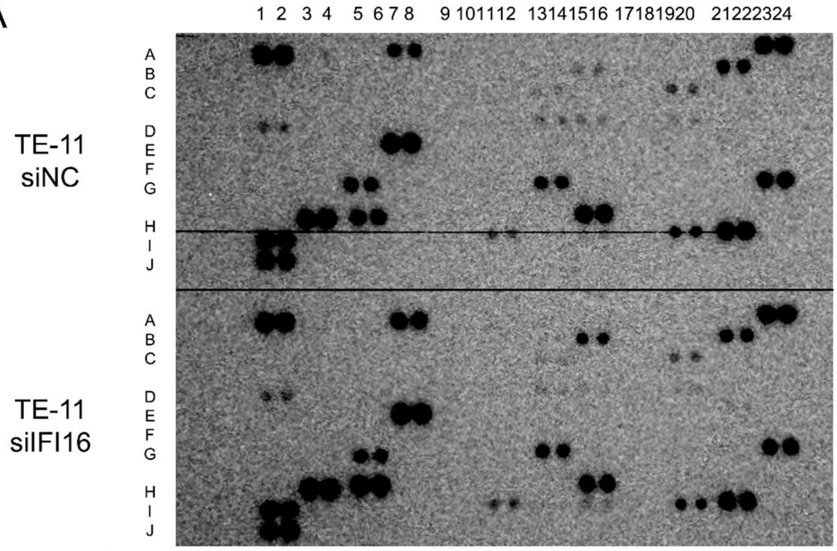

B

Coordinate	Analyte / Control	Coordinate	Analyte / Control	Coordinate	Analyte / Control	Coordinate	Analyte / Control
A1, A2	Reference Spots	D1, D2	GROα	G1, G2	Leptin	J1, J2	Reference Spots
A3, A4	Adiponectin	D3, D4	Growth Hormone	G3, G4	LIF	J3, J4	
A5, A6	Apolipoprotein A-	D5, D6	HGF	G5, G6	Lipocalin-2	J5, J6	Vitamin D BP
A7, A8	Angiogenin	D7, D8	ICAM-1	G7, G8	MCP-1	J7, J8	CD31
A9, A10	Angiopoietin-1	D9, D10	IFN-γ	G9, G10	MCP-3	J9, J10	TIM-3
A11, A12	Angiopoietin-2	D11, D12	IGFBP-2	G11, G12	M-CSF	J11, J12	VCAM-1
A13, A14	BAFF	D13, D14	IGFBP-3	G13, G14	MIF	J13, J14	
A15, A16	BDNF	D15, D16	IL-1α	G15, G16	MIG	J15, J16	
A17, A18	Complement Component C5/C5a	D17, D18	IL-1β	G17, G18	MIP-1α/MIP-1β	J17, J18	
A19, A20	CD14	D19, D20	IL-1ra	G19, G20	MIP-3α	J19, J20	
A21, A22	CD30	D21, D22	IL-2	G21, G22	MIP-3β	J21, J22	
A23, A24	Reference Spots	D23, D24	IL-3	G23, G24	MMP-9	J23, J24	Negative Controls
B1, B2		E1, E2	IL-4	H1, H2	Myeloperoxidase		
B3, B4	CD40 ligand	E3, E4	IL-5	H3, H4	Osteopontin		
B5, B6	Chitinase 3-like 1	E5, E6	IL-6	H5, H6	PDGF-AA		
B7, B8	Complement Factor D	E7, E8	IL-8	H7, H8	PDGF-AB/BB		
B9, B10	C-Reactive Protein	E9, E10	IL-10	H9, H10	Pentraxin 3		
B11, B12	Cripto-1	E11, E12	IL-11	H11, H12	PF4		
B13, B14	Cystatin C	E13, E14	IL-12 p70	H13, H14	RAGE		
B15, B16	Dkk-1	E15, E16	IL-13	H15, H16	RANTES		
B17, B18	DPPIV	E17, E18	IL-15	H17, H18	RBP-4		
B19, B20	EGF	E19, E20	IL-16	H19, H20	Relaxin-2		
B21, B22	EMMPRIN	E21, E22	IL-17A	H21, H22	Resistin		
B23, B24		E23, E24	IL-18 Bpa	H23, H24	SDF-1α		
C1, C2		F1, F2	IL-19	I1, I2	Serpin E1		
C3, C4	ENA-78	F3, F4	IL-22	I3, I4	SHBG		
C5, C6	Endoglin	F5, F6	IL-23	I5, I6	ST2		
C7, C8	Fas Ligand	F7, F8	IL-24	I7, I8	TARC		
C9, C10	FGF basic	F9, F10	IL-27	I9, I10	TFF3		
C11, C12	FGF-7	F11, F12	IL-31	I11, I12	TfR		
C13, C14	FGF-19	F13, F14	IL-32	I13, I14	TGF-α		
C15, C16	Flt-3 Ligand	F15, F16	IL-33	I15, I16	Thrombospondin-1		
C17, C18	G-CSF	F17, F18	IL-34	I17, I18	TNF-α		
C19, C20	GDF-15	F19, F20	IP-10	I19, I20	uPAR		
C21, C22	GM-CSF	F21, F22	I-TAC	I21, I22	VEGF		
C23, C24		F23, F24	Kallikrein 3	I23, I24			

Figure S1. (A) Cytokine array results of supernatants from TE-11 cells transfected with siNC (upper panel) and siIFI16 (lower panel). (B) Coordinates of the cytokine array.

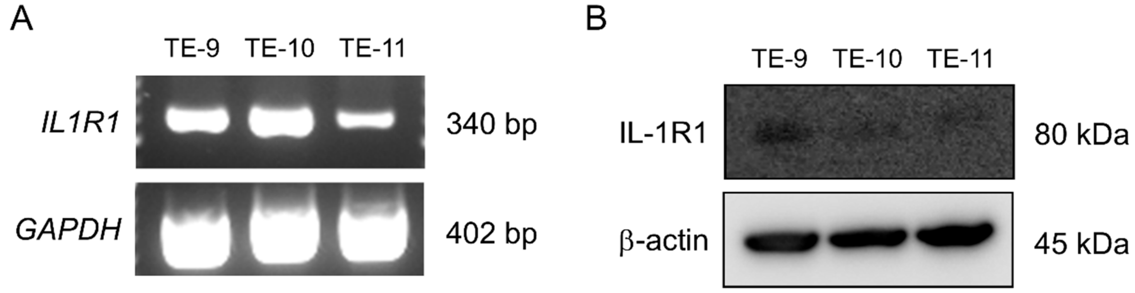

Figure S2. Expression levels of IL-1R1 in ESCC cells. (A, B) The expression of IL-1R1 was confirmed by RT-PCR (A) and Western blotting (B*). GAPDH* and β-actin was used as internal control in RT-PCR and Western blotting, respectively.

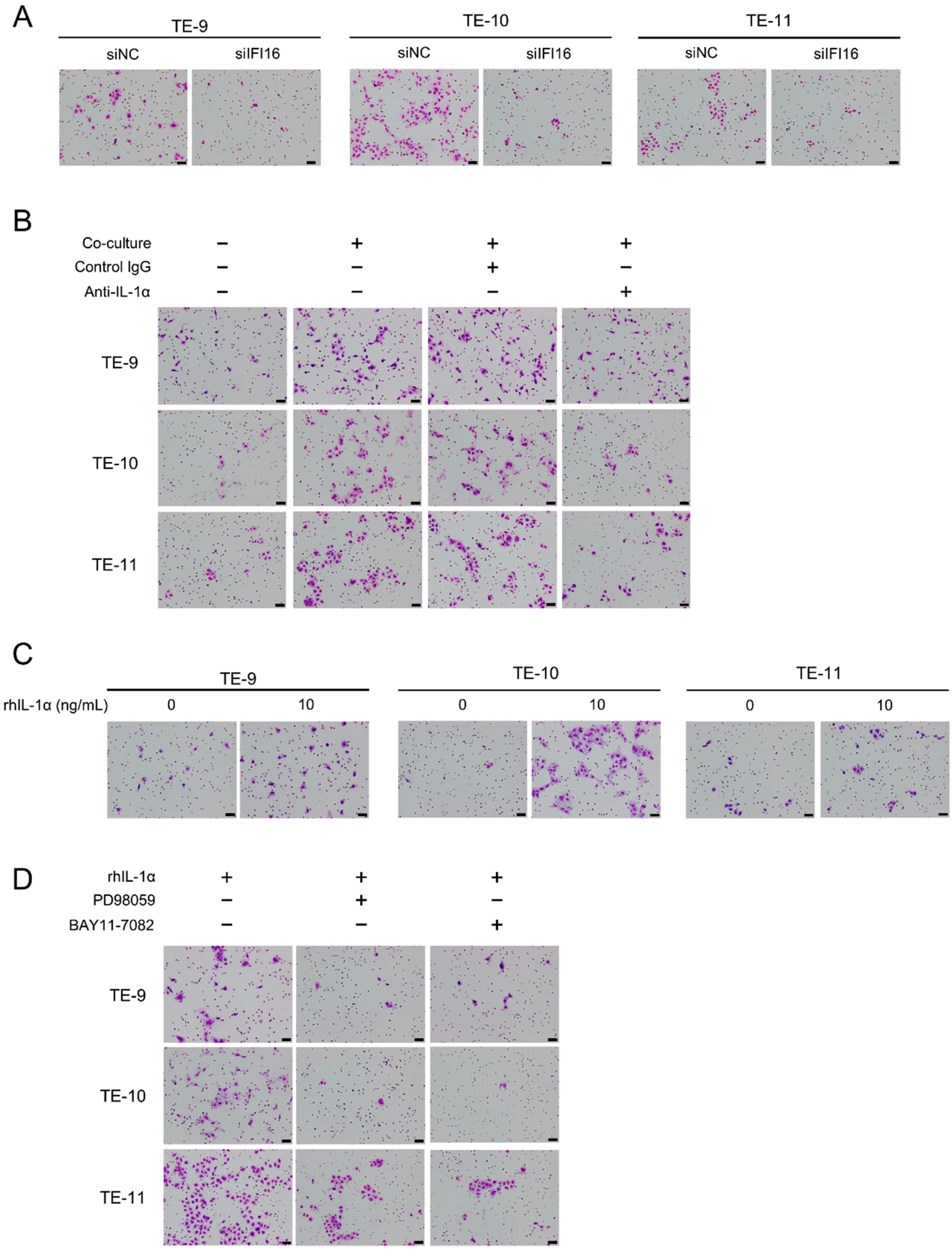

Figure S3. Representative images (×200) of migration assays in Figures 2E, 3F, 4C, and 4G, corresponding to Figures S3A–D, respectively. Scale bar: 50 µm.

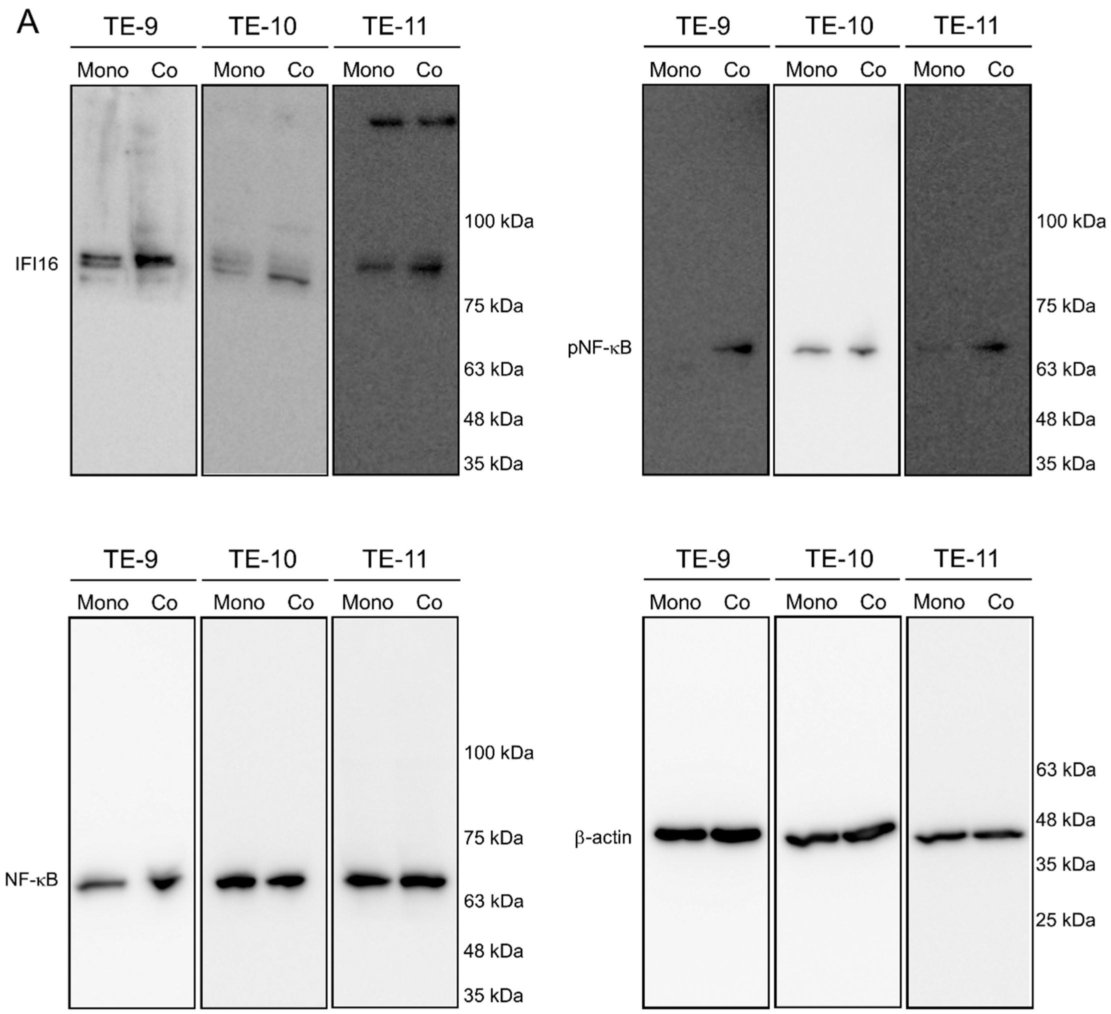
A
TE-9
TE-10
TE-11
Mono
Co
IFI16
100 kDa
75 kDa
63 kDa
48 kDa
35 kDa
pNF-κB
NF-κB
β-actin
25 kDa

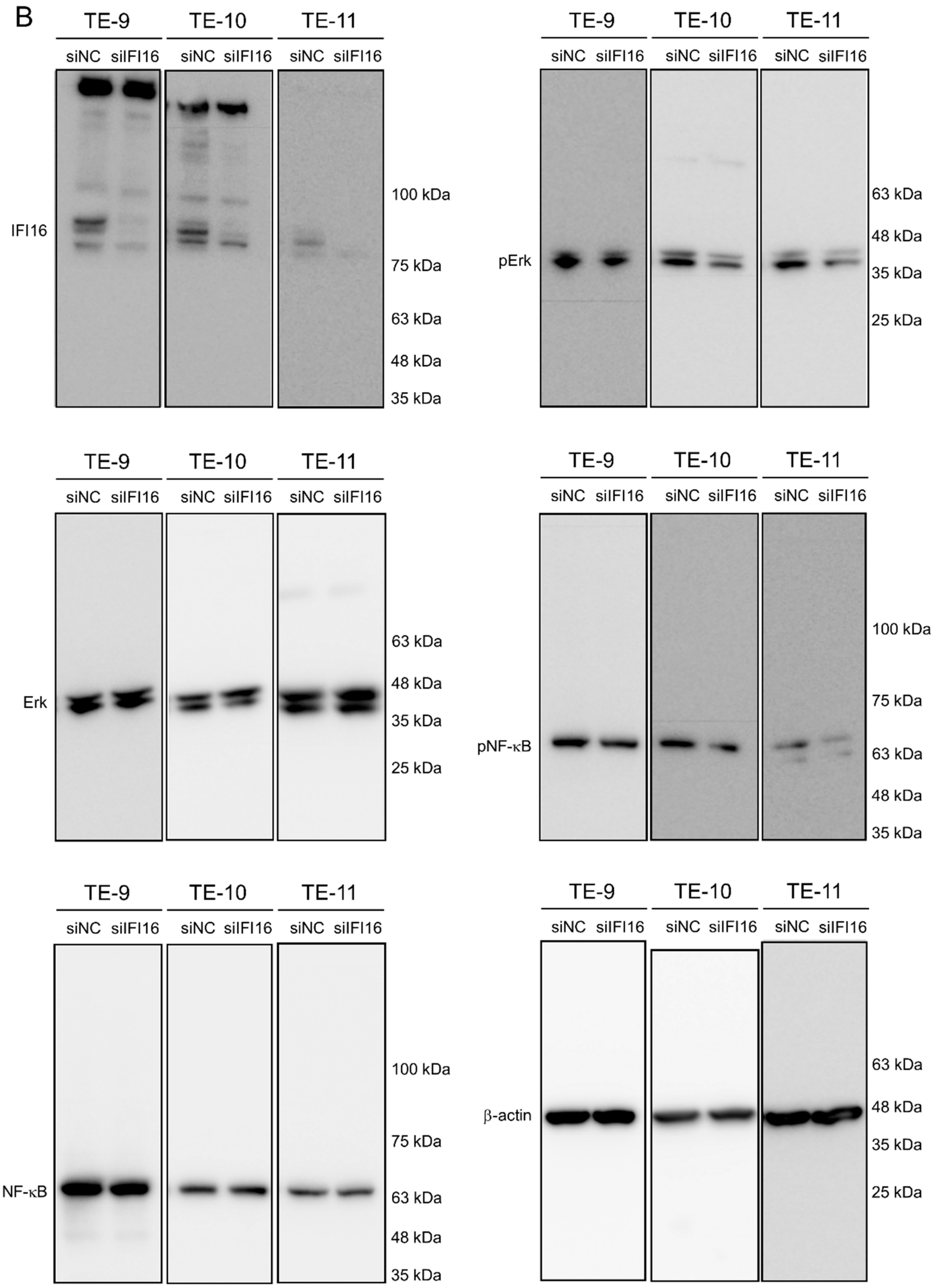
B
TE-9
TE-10
TE-11
siNC
siIFI16
IFI16
100 kDa
75 kDa
63 kDa
48 kDa
35 kDa
pErk
25 kDa
Erk
pNF-κB
NF-κB
β-actin

C

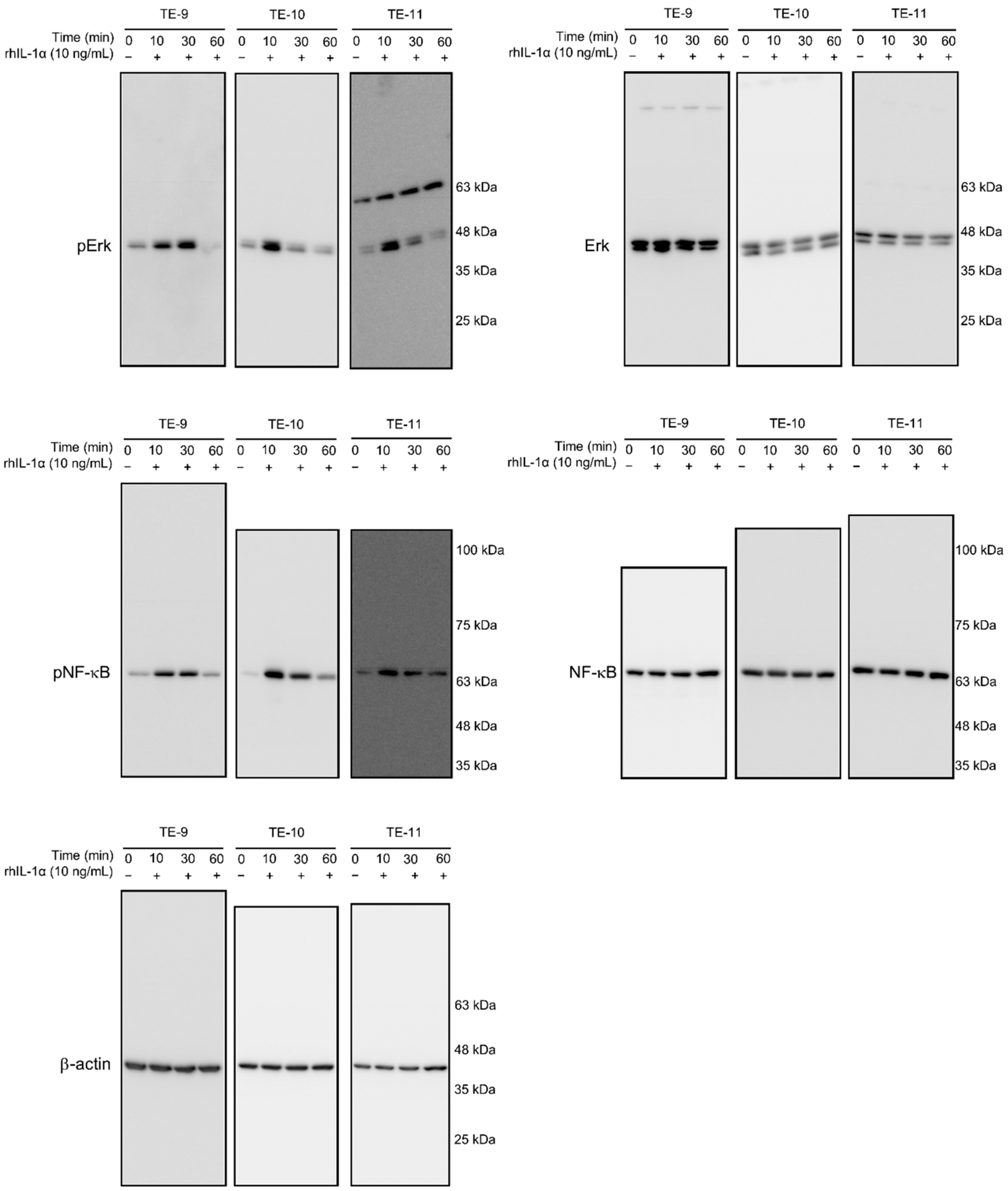
TE-9
TE-10
TE-11
Time (min) 0 10 30 60 0 10 30 60 0 10 30 60
rhIL-1α (10 ng/mL) – + + + – + + + – + + +
pErk
63 kDa
48 kDa
35 kDa
25 kDa
TE-9
TE-10
TE-11
Time (min) 0 10 30 60 0 10 30 60 0 10 30 60
rhIL-1α (10 ng/mL) – + + + – + + + – + + +
Erk
63 kDa
48 kDa
35 kDa
25 kDa
TE-9
TE-10
TE-11
Time (min) 0 10 30 60 0 10 30 60 0 10 30 60
rhIL-1α (10 ng/mL) – + + + – + + + – + + +
pNF-κB
100 kDa
75 kDa
63 kDa
48 kDa
35 kDa
TE-9
TE-10
TE-11
Time (min) 0 10 30 60 0 10 30 60 0 10 30 60
rhIL-1α (10 ng/mL) – + + + – + + + – + + +
NF-κB
100 kDa
75 kDa
63 kDa
48 kDa
35 kDa
TE-9
TE-10
TE-11
Time (min) 0 10 30 60 0 10 30 60 0 10 30 60
rhIL-1α (10 ng/mL) – + + + – + + + – + + +
β-actin
63 kDa
48 kDa
35 kDa
25 kDa

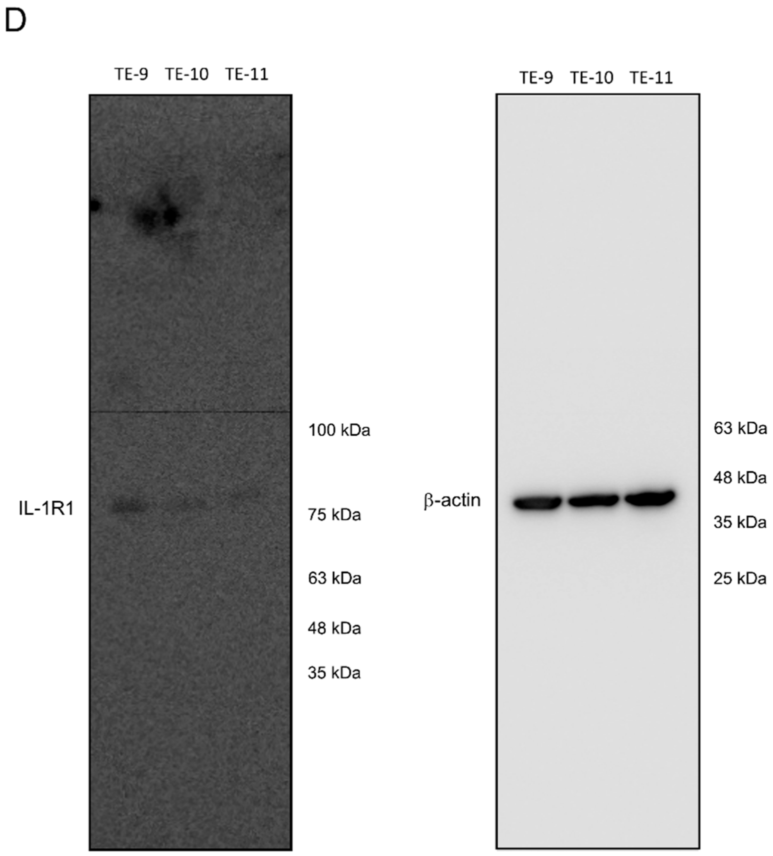

Figure S4. Raw images of Western blotting from Figures 1B, 2B, 4D, and S2B, corresponding to Figures S4A–D, respectively. The markers were not visualized in the raw membrane data because three colored pre-stained markers without chemiluminescent substances were used.
